# Mesenchymal Stromal Cells-Derived Extracellular Vesicles as Potential Treatments for Osteoarthritis

**DOI:** 10.3390/pharmaceutics15071814

**Published:** 2023-06-25

**Authors:** Shunling Yuan, Guangfeng Li, Jinbo Zhang, Xiao Chen, Jiacan Su, Fengjin Zhou

**Affiliations:** 1 School of Pharmacy, Naval Medical University, Shanghai 200433, China; sl_yuan@foxmail.com (S.Y.); jbzhangs@foxmail.com (J.Z.); 2 Department of Orthopedics Trauma, Shanghai Zhongye Hospital, Shanghai 200941, China; dlgf84@163.com; 3 Department of Pharmacy, Tianjin Rehabilitation Center of Joint Logistics Support Force, Tianjin 300110, China; 4 Department of Orthopedics Trauma, Shanghai Changhai Hospital, Naval Medical University, Shanghai 200433, China; sirchenxiao@126.com; 5 Institute of Translational Medicine, Shanghai University, Shanghai 200444, China; 6 Institute of Advanced Interdisciplinary Materials Science, Shanghai University, Shanghai 200444, China; 7 Organoid Research Center, Shanghai University, Shanghai 200444, China; 8 Department of Orthopaedics, Honghui Hospital, Xi’an Jiao Tong University, Xi’an 710000, China

**Keywords:** extracellular vesicles, mesenchymal stromal cells, osteoarthritis, degenerative joint diseases

## Abstract

Osteoarthritis (OA) is a degenerative disease of the joints characterized by cartilage damage and severe pain. Despite various pharmacological and surgical interventions, current therapies fail to halt OA progression, leading to high morbidity and an economic burden. Thus, there is an urgent need for alternative therapeutic approaches that can effectively address the underlying pathophysiology of OA. Extracellular Vesicles (EVs) derived from mesenchymal stromal cells (MSCs) represent a new paradigm in OA treatment. MSC-EVs are small membranous particles released by MSCs during culture, both in vitro and in vivo. They possess regenerative properties and can attenuate inflammation, thereby promoting cartilage healing. Importantly, MSC-EVs have several advantages over MSCs as cell-based therapies, including lower risks of immune reactions and ethical issues. Researchers have recently explored different strategies, such as modifying EVs to enhance their delivery, targeting efficiency, and security, with promising results. This article reviews how MSC-EVs can help treat OA and how they might work. It also briefly discusses the benefits and challenges of using MSC-EVs and talks about the possibility of allogeneic and autologous MSC-EVs for medical use.

## 1. Introduction

**Figure 1:**
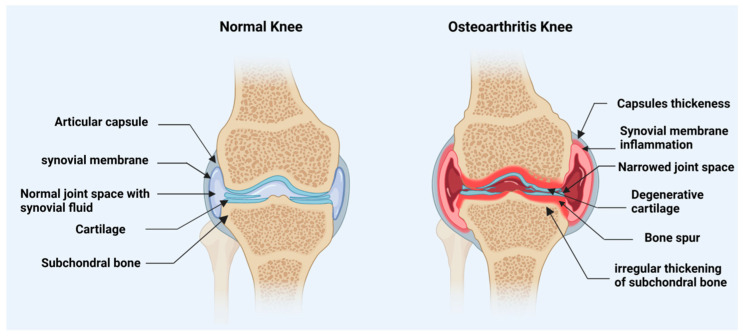
An illustration of a healthy knee joint compared to an osteoarthritic knee. Inflammation of the synovial membrane, bone spurs, and cartilage loss are hallmarks of osteoarthritis. Created with BioRender (BioRender.com, 20 February 2023).

Osteoarthritis (OA) is a type of chronic joint disease that slowly damages the cartilage covering bones and causes constant pain (Figure 1). In light of the accelerating aging of the global population. By 2019, OA was the fifteenth most common cause of disability and affected about 240 million people around the world [1,2].

Although OA can occur in any synovial joint, it most commonly affects load-bearing bones, including hands, knees, and big toes, leading to a significant decline in the well-being of patients as well as challenges for the patient’s relatives and society [3]. Joint damage can be caused by many risk factors, including age, gender, obesity, genetic traits, and serious joint trauma. Aging is considered a key factor that leads to OA [4]. 

Current therapies for OA can be divided into several types: non-pharmacological, like exercise and physical therapy [1]; pharmacological strategies, such as opioids and anti-inflammatory drugs, including nonsteroidal anti-inflammatory drugs (NSAIDs) [5]; and surgical options like joint substitution [6]. However, these therapies are unable to slow down OA deterioration or promote the healing of damaged cartilage [7]. In recent years, cell-based therapies have become a hopeful alternative for OA treatment [8]. Mesenchymal stromal cells (MSCs) are pluripotent cells able to renew themselves and differentiate into different cell types, which makes them appealing for healing OA joints that are damaged, while the immune rejection and tumorigenicity of cell therapy are unavoidable [9]. Hence, novel and effective treatments are urgently needed.

Extracellular vesicles (EVs) are tiny vesicles that can transfer signals between cells and help maintain balance and function in tissues and organs [10]. EVs from MSCs (MSCs-EVs) share some of the beneficial features of MSCs, while having some advantages over the donor cells, including a lower risk of immune rejection and easier collection and storage [11,12]. MSC-EVs have been reported to modulate inflammation and stimulate cartilage repair, which makes them promising candidates for OA treatment [13,14].

In the review, we discuss how MSC-EVs from various sources can help treat OA and how they might work, outline the limits of MSC-EVs in clinical application, and propose potential applications in drug delivery. Such information provides a valuable reference for designing novel therapeutic strategies for OA treatment.

## 2. Pathophysiology of OA

OA is believed to be a disorder that affects the entire joint and has been a tremendous burden for the health care system globally [2,15]. The causes of OA are complex and involve various factors, such as mechanical stress, inflammation, and metabolic changes, resulting in damage and dysfunction of the synovial joint. Based on its etiology, OA can be divided into primary and secondary OA: As of yet, it is unclear what causes primary OA, but the role of mechanical and biochemical factors is mainly considered; Secondary OA is commonly caused by the following factors: traumatic arthritis, infection, and inflammatory reactions, which are relatively well understood [16].

The disease is an active dynamic alteration arising from an imbalance between the repair and destruction of joint tissues, not a passive degenerative disease as commonly described [4]. The pathogenesis of OA can be summarized as (i) dysregulation of cartilage matrix synthesis and catabolism; (ii) loss of cartilage cushioning due to damage to the subchondral bone plate; and (iii) focal inflammation within the joint [17].

Cartilage degeneration is the characteristic pathological change and the most basic pathological change of OA [18]. Collagen type II is the primary structural protein of articular cartilage. Together with other types of collagen and non-collagenous proteins, it forms a meshwork structure that provides the cartilage with tensile strength and maintains cartilage stability. Cartilage resists compression because it draws water into itself through proteoglycans and other proteoglycans that are implanted within this meshwork. When the external chemical and mechanical environment changes, chondrocytes can modulate the structural and biochemical composition of cartilage to adapt to this change [19,20]. As OA progresses, chondrocytes produce a variety of inflammatory response proteins, such as matrix metalloproteinase (MMP), novel metalloproteinases (ADAMTS), IL-1β, TNF-α, and other inflammation-related factors [21]. Among these, metalloproteinases 1, 3, and 13, and novel metalloproteinases 4 and 5 degrade proteoglycans and collagen, leading to cartilage destruction in OA [22].

Under the hyaline cartilage and cement line is a bony layer called the subchondral bone. This layer has two parts: a plate of cortical bone and a network of trabecular bone [7]. The cortical bone plate is a solid lamellar structure that provides mechanical support to the articular cartilage and cushions the stress load, while the latter is a porous structure with abundant vascular and nerve penetration that responds to local stress changes and provides nutritional support to the cartilage. 

During OA, both the cortical bone plate and trabecular bone undergo significant structural and compositional changes, which may occur earlier than the articular cartilage and worsen the degradation of the articular cartilage from the bottom up [23]. In the early stages of OA, there is increased bone resorption activity due to accelerated bone conversion of the subchondral bone, which is manifested by the reduced thickness of the subchondral bone plate, increased loss of bone trabecular, and reduced mechanical strength [24]. In the later stages of OA, bone formation becomes progressively more dominant, as evidenced by an increase in the thickness of the subchondral bone plate and sclerosis of the cancellous bone. At the same time, there is progressive damage to the non-calcified cartilage, which is accompanied by the thickening of the tidemark replication [25]. Abnormal subchondral bone reconstruction and disruption of cartilage integrity interact with each other and together lead to OA.

An early feature of OA is synovitis. Synovial membranes cover the inner surface of the joint capsule around the joints with loose connective tissue. It synthesizes and secretes synovial fluids such as hyaluronic acid and lubricant to reduce friction on the joint surface and prevent adhesion, as well as provide nutrients to cartilage and excrete waste products. During OA, synovial cells are activated, and cells proliferate, hypertrophy, and release inflammatory mediators and degradative enzymes [26]. These inflammatory mediators stimulate the synthesis of metalloproteinases by the chondrocytes and accelerate the degeneration of articular cartilage. Synovial cells themselves can also induce angiogenesis and increase the synthesis of inflammatory mediators and degradative enzymes, as described above, creating a vicious cycle of positive feedback that is an important driver of the development of osteoarthritis [27].

## 3. Biological Characteristics of EV

Using EVs to treat OA has become a popular topic in biomedicine in the last ten years. EVs can carry drugs, genes, and proteins to target cells and tissues, making them ideal for curing and preventing various diseases. Here, we give an overview of EV biogenesis, composition, and application, in order to better understand their applications in OA.

### 3.1. Production and Release of EVs

**Figure 2:**
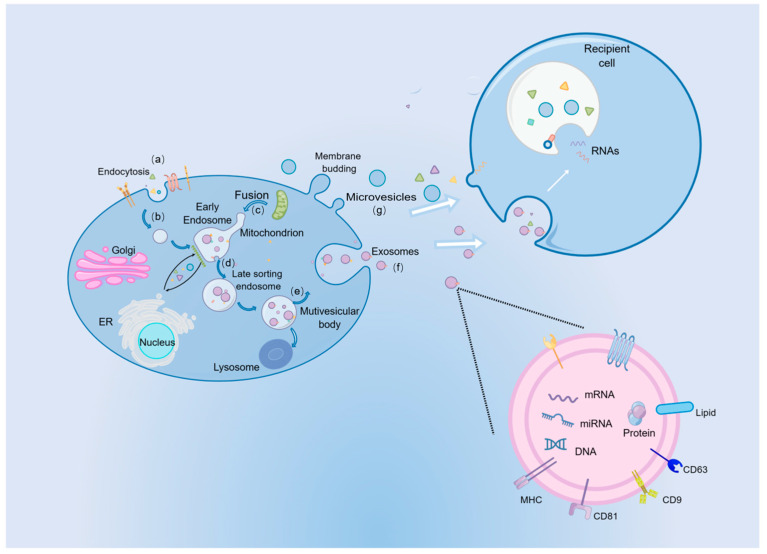
Biogenesis, secretion, and structure of EVs. (a) Through endocytosis, extracellular components and proteins enter cells. (b) Budding of the cell membrane occurred on the luminal side. (c) Following the fusion of the bud with the endoplasmic reticulum, golgi apparatus, and mitochondria, the early sorting endosomes formed. (d) Consequently, early sorting endosomes turned into late sorting endosomes. (e) The multivesicular body is formed with intraluminal vesicles containing various cargos. (f) In the process of exocytosis, intraluminal vesicles are released into the cytoplasm as exosomes. (g) The microvesicles are formed by directly pinching off the cell membrane. By Figdraw.

Extracellular Vesicles (EVs) refer to nanoparticles that are released by cells into the extracellular space, playing an important role in cell-cell communication [28]. EVs are classified into several major subtypes based on their biogenesis: microvesicles, exosomes, and large EVs, including apoptotic bodies and oncosomes. The sizes, contents, and formation mechanisms of the three types of extracellular vesicles are all different (Figure 2). Here, we focus on the first two classes of EVs, which are released under the normal physiological state of cells. Exosomes range from 30 to 100 nm in diameter and are formed by the fusion of endosomal compartments with the plasma membrane. Conversely, microvesicles (100–1000 nm in diameter) develops from direct pinching off of the cell membrane following budding. It is known that EVs are present in a number of biological fluids, including blood, urine, and breast milk, as well as amniotic fluid-conditioned medium for cell culture [29]. It was once believed that cells used these structures to dispose of cellular waste and transport metabolic products; however, accumulating evidence indicated that EVs are greatly involved in cell-to-cell communication as a natural endogenous nanocarrier. By transferring membrane receptors, proteins, lipids, and RNAs [30], EVs can participate in cell signaling and intercellular communication. The cargo that small EVs carry and their interaction with recipient cells determine their function [31].

### 3.2. Characteristics of MSC-Derived EVs

MSCs are progenitors for the mesoderm lineage with very high differentiation potential, deriving from a variety of sources like bone marrow, adipose tissue, and synovium [32]. To ensure the integrity and unambiguous identification of human MSCs, the International Society for Cell Therapy (ISCT) has suggested some basic criteria: (1) MSCs must adhere to plastic culture under standard conditions; (2) As MSCs lack a single peculiar marker, they are identified and categorized based on their specific characteristics and detailed expression pattern [33]. However, accumulating evidence indicates that heterogeneous sources of MSCs have various surface markers [34]. It is possible to use these MSC subpopulations for specific medical conditions, according to their unique features, to improve therapeutic outcomes.

Easily cultivated and proliferating in vitro, MSCs have been studied extensively for clinical purposes and have proven effective in treating osteoarthritis [35,36,37,38,39]. Nevertheless, MSC therapy does have some aspects that merit consideration. For one thing, MSCs may become differentiated and even aged over time when cultured in vitro with increasing passages, thus altering their regulatory and therapeutic functions [40]. Additionally, it is not clear if MSCs are capable of targeting or residing at damaged sites in vivo. It is evident that the natural targeting of delivered MSCs to specific tissues is very low, as only less than 1% of them reach their destination [41]. In addition, the risk of potential tumorigenicity related to MSC-based therapies cannot be ignored [42]. Moreover, the storage and transportation of MSCs is one of the key concerns for MSC-mediated tissue regeneration.

According to recent research, researchers have found that paracrine secretion is an essential component for MSCs’ role in bone regeneration, and EVs are a key mediator of this process [43,44]. MSC-derived EVs express EV surface markers CD63, CD9, and CD81 [45], as well as mesenchymal stromal cell surface markers [46]. There are variable phenotypes and roles for MSC-EVs in accordance with donor cells [47]. The MSC-EVs’ molecular basis for function is a charge of nucleic acids, proteins, and lipids, therefore delivering information and communicating with target cells [48]. Their high safety profile, low immunogenicity, and tumorgenicity make them excellent candidates for a new bone regenerative strategy. 

### 3.3. Features of Various MSC-Derived Extracellular Vesicles

Due to their diversity, EV cargo could contain multiple bioactive molecules, including proteins, lipids, and miRNAs, resulting in different traits of EVs. A bioactive molecule from various sources might be responsible for MSCs’ different therapeutic effects. Here, we summarize the EVs for OA treatment derived from different MSCs (Table 1).

#### 3.3.1. BMSC-EVs

The evidence is growing that EVs derived from BMSCs, which maintain the excellent traits of BMSCs, may have a stronger role in cartilage repair. BMSC-EVs can not only affect cell survival, such as apoptosis, proliferation, and migration but also control many pathological processes in OA. Recent studies show that BMSC-EVs can induce cartilage regeneration and sclerosed subchondral bone repair. Li et al. [50] found that MSCs-circHIPK3-EVs promote the expression of MYH9 in chondrocytes. MSCs-circHIPK3-EVs attenuated chondrocyte injury and inhibited cartilage degradation by positively regulating MYH9 expression via binding to miR-124-3p. It may be possible to develop a new treatment for OA based on the results of this study. The impact of BMSC-EVs on cartilage damage and pain behavior in mice with OA induced by sodium iodoacetate was studied by He and his colleagues [58]. Their study evaluated changes in cartilage and the dorsal root ganglion (DRG) of mice treated with BMSC-EVs. They discovered that BMSC-EVs may alleviate pain by suppressing the expression of CGRP and iNOS in the DRG, thus relieving the inflammatory and neuropathic pain in OA rats simultaneously. Moreover, BMSC-EVs can promote IL-1β-treated chondrocytes proliferation and significantly inhibit apoptosis by inactivating the Wnt/β-Catenin pathway, demonstrating that BMSC-EVs are effective at protecting chondrocytes under inflammatory conditions [59]. In conclusion, BMSC-EVs are an applicable method for OA therapy.

#### 3.3.2. ADSC-EVs

EVs derived from ADSCs can also be involved in OA treatment. It has been proved that ADSC-EVs can control joint regeneration and inflammation. According to Woo et al. [60], ADSC-EVs can promote the physiological activities of human OA chondrocytes as well as regulate the expression of degradation-related molecules in vitro. Moreover, in vivo, ADSC-EVs effectively prevented OA in sodium mono iodoacetate rats and OA mice treated with surgical damage by intra-articular injection. Wu et al. [57] found that miR-100 could regulate AKT/mTOR signaling by targeting the 3′-UTR of mTOR in cell autophagy, and mTOR/autophagy signaling pathway plays a key role in OA progression. With treat of MSCIPFP-EVs enriched in miR-100-5p, chondrocytes underwent apoptosis reduction, while matrix synthesis was enhanced. Furthermore, miR-100-5p has been found to bind mTOR’s 3’ untranslated region (3’UTR) and significantly enhance autophagy levels in chondrocytes via mTOR inhibition. ADSCs could promote repair and regeneration by secreting paracrine trophic factors [61]. In the light of Miguel Tofiño-Vian et al. [62], ADSC-EVs modulate chondrocyte metabolism to counteract the effects of IL-1β, such as the secretion of cytokines and other inflammation-related factors.

#### 3.3.3. SMSC-EVs

With remarkable proliferative and chondrogenic potential, SMSCs can impart the possibility of repairing cartilage injuries by accelerating the proliferation of chondrocytes. It has been demonstrated that SMSC-EVs can effectively stimulate cartilage repair as well as inhibit OA. Lu et al. [63] revealed how SMSC-EVs act as natural carriers of miR-31. MiR-31 has been identified to downregulate in OA patients, which is negatively correlated with KDM2A [55]. With miR-30 enriched in SMSC-EVs, SMSC-EVs can dramatically inhibit apoptosis and the inflammatory response of OA, and the expression of KDM2A was also decreased under the action of SMSC-EVs. To explore the fundamental mechanism, miR-31 carried by SMSC-EV targets KDM2A to activate the E2F1/PTTG1 Axis, leading to the stimulation of chondrocytes’ proliferation and migration. Moreover, Tao et al. [54] discovered that alternate Wnt signaling pathways could activate YAP, and also inhibited extracellular matrix (ECM) synthesis by inhibiting SOX9 at the same time. To understand how these phenomena occur, researchers enriched mir-140-5p in SMSCs and collected their EVs as natural transporters. This study illustrated that SMSCs containing miR-140-5p exerted an inhibitory effect on Ras-related protein (RalA) to promote SOX9 inhibition by the alternative Wnt-YAP/TAZ signaling pathway, thus restoring extracellular matrix (ECM) secretion. In this report, SMSC-EVs have been uncovered as potential therapeutic approaches for treating OA via signaling pathway regulation.

#### 3.3.4. EVs derived from other MSCs

With outstanding self-renewal capacity, MSCs from other tissues also are widely applied in OA treatment. Having the advantages of adequate tissue supply, rapid growth capacity, and excellent differentiation capacity, Umbilical cord MSCs (UMSCs) are ideal alternatives in cartilage therapy [64].

In recent explorations, UMSCs-EVs have been shown to promote cartilage regeneration [65]. Researchers found that UMSCs-EVs could enhance chondrocyte migration and proliferation, stimulating chondrogenesis. MiR-23a-3p was the most enriched miRNA in UMSCs-EVs according to the miRNA microarray. After transferring miR-23a-3p, UMSCs-EVs can benefit cartilage defect treatment by activating the PTEN/AKT signaling pathway. In addition, UMSCs-EVs have also been proven to induce M2 macrophage polarization and inflammatory inhibition [14]. Moreover, induced pluripotent stem cells (iPSC) are another source for MSC-EVs, which can differentiate into iPSC-derived MSCs (iMSCs). According to a recent study, iPSC-EVs can stimulate the proliferation of primary human chondrocytes and suppress cell senescence by increasing the expression of collagen II and p21 in an IL-1β-induced in vitro OA model. Moreover, iPSC-EVs inhibited OA progression and cartilage destruction in a rabbit ACLT model [66].

## 4. Therapeutic Application of MSC-EV in OA

### 4.1. Immunomodulatory Effect

The immune system is generally thought to function in part to defend the body against viruses and other external threats. Tissue healing is influenced by the dynamic balance between inflammatory and anti-inflammatory factors, ensuring cartilage maintains a normal physiological state. It has been reported that immune cells like macrophages, neutrophils, and synovium cells release amounts of pro-inflammatory factors when stimulated by internal and external factors, causing matrix destruction and articular cartilage degeneration [67,68]. Hence, OA therapy must control the pro-inflammatory milieu in the cartilage microenvironment.

The characterization of macrophage phenotypes has been previously classified into proinflammatory or anti-inflammatory types, based on their M1 or M2 phenotypes. The M1 macrophages have been identified to produce cytokines such as IL-1, IL-6, and IL-12, which can trigger inflammation upon stimulation by IFN-γ, TNF-α or other molecules that have an association with inflammation [69]. On the other hand, activation of M2 macrophages is found to contribute to the suppression of inflammation and tissue repair [70].

The T cells that infiltrate the synovial fluid of patients with Osteoarthritis (OA) are classified into effector T cells, including cytotoxic T cells and helper T cells, as well as regulatory T cells [71]. The role of both cytotoxic T cells (T_C_ cells) and helper T cells (T_H_ cells) in the pathogenesis of OA has been established due to their increased levels in the peripheral blood, synovial membranes, and fluid of OA patients [72]. Inflammatory cytokines produced by T_H_ 1, T_H_ 9, and T_H_ 17 exacerbate joint inflammation and cause tissue damage [73]. Tc cells contribute to OA biology, although the mechanisms remain unclear. Regulatory T cells release cytokines that hinder immune responses and maintain immune tolerance. In the pathogenesis of OA, a reduction in Tregs activity is observed [73]. The alteration of the T_H_ 17/Treg ratio leads to an exacerbated form of arthritis [74].

Recent studies suggest that B cells, are involved in the pathogenesis of osteoarthritis through multiple mechanisms. B cells can produce various antibodies and cytokines that contribute to the inflammation and osteoclast-mediated bone resorption in osteoarthritis affected joints [75]. They can also interact with other immune cells, such as T cells and dendritic cells, to enhance the inflammatory response [76]. Moreover, B cells have been found to be present in synovial fluid from osteoarthritis patients. Therefore, the involvement of B cells in multiple aspects of osteoarthritis pathogenesis highlights them as a potential therapeutic target for the treatment of this debilitating condition.

MSC-EVs have recently been examined as regulators of both innate (macrophages) and adaptive immune cells (B and T cells). As reported by Zhang et al., the infiltration of M1 macrophages into OA cartilage defects can be strongly inhibited by MSC-EVs, while the infiltration of M2 macrophages can be enhanced. Meanwhile, a reduction in pro-inflammatory synovial cytokines, like IL-1β and TNF-α was also confirmed [77]. These resulted in an overall decrease in OA inflammation. In osteoarthritis, T-cell-mediated inflammation can contribute to the progression of disease and the exacerbation of symptoms such as joint pain and stiffness [71]. MSC-EVs can inhibit the activation and proliferation of T cells, leading to a decrease in pro-inflammatory cytokines (such as IL-17A, IL-21, IL-22, and IL-2) that contribute to the pathogenesis of osteoarthritis [78,79]. B cells are key cellular components of the immune system and play a role in OA pathogenesis. Recent studies have demonstrated that MSC-EVs can regulate B cell function. In particular, MSC-EVs can suppress the activation of B cells, decrease their proliferation, and induce apoptosis [80,81]. However, an investigation, distinct from preceding studies, has documented the negligible or absence of functional impacts exhibited by extracellular vesicles (EVs) on B-cells [82]. The precise mechanisms underlying the involvement of EVs in MSC-facilitated regulation of B-cell immunity warrant further investigation.

### 4.2. Promoting Chondrocyte Survival

In the OA cartilage microenvironment, as a result of inflammation, injured cartilage suffers from cell death, matrix degradation, and ultimately loss of structure and function. The apoptosis of chondrocytes is linked to cartilage degeneration, and MSCs-EVs have also been reported to promote these cells’ survival [77,83].

Chondrocyte activity and matrix composition are altered in OA, which is the only cell type in articular cartilage that can balance the synthesis and breakdown of extracellular matrix. So, the migration and proliferation of chondrocytes are also involved in OA therapy, both of which are suppressed during OA progression. Research shows that MSC-EVs can alter adhesion, proliferation, and migration related proteins within chondrocytes [77,84,85]. EVs change the expression levels of genes such as Sox9, and Col II to promote chondrogenesis, and this effect can be effectively reversed in the presence of ICG-001, an antagonist of Wnt/β-catenin signaling [86]. MSC-EVs are known to improve s-GAG synthesis and suppress NO and MMP-13 production in TMJ-OA models. These effects prevented proliferation and migration declines. Another benefit of EVs from MSC is that they can enhance the repair of cartilage in chondrocytes by making more GAG and raising the level of COL II protein. As a result, these EV proteins increase the stability of the ECM and actin cytoskeletal dynamics, which in turn affect chondrocyte proliferation and migration in an indirect manner [13]. Another way to promote chondrocyte survival is by inhibiting chondrocytes decreasing caused by cell death and apoptosis, MSC-EVs can improve chondrocyte cell performance through related signaling pathways inhibition and autophagy promoters [63,87,88].

### 4.3. Promoting ECM Synthesis

The ECM plays an important role in cartilage structure, two key components of which are collagen II and proteoglycans. During OA pathology, the inflammatory micro-environment tends to release inflammation-related factors such as MMP-13, and ADAMTS-5, inducing a reduction in COL II levels and proteoglycan levels within OA joints, which results in the degeneration of cartilage [89]. Recent research suggests that MSC-EVs can boost anabolic gene expression and help rebuild the cartilage matrix [60,90,91]. Tao et al. [54] isolated EVs from SMSCs. When OA chondrocytes were treated with Evs enriched miR-140-5p, the function of SOX9 was repaired via regulation of RalA, and ECM secretion was successfully restored.

Overall, the effect of MSC-Evs on immune system during OA therapy appears to be immunosuppressive, reducing the activity of these cells and potentially contributing to the anti-inflammatory and chondroprotective properties of MSC-Evs based therapies (Table 2).

## 5. The Role of MSC-EVs as Drug-Delivery Vesicles

**Figure 3:**
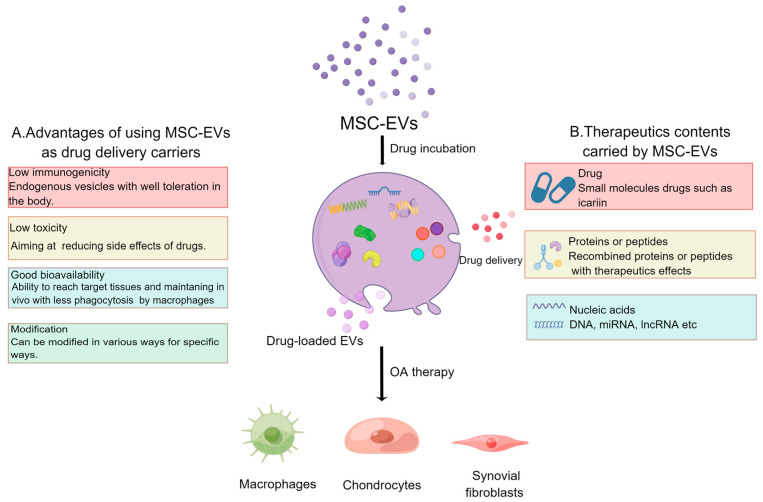
MSC-EVs can be used as drug-delivery systems. (**A**) Advantages of using MSC-EVs as a drug-delivery system. (**B**) Therapeutic contents that can be delivered via milk EVs. By Figdraw.

Currently, the primary method of treating osteoarthritis (OA) involves drug intervention. The most common routes of administration include oral or topical/transdermal applications. However, due to the localized nature of OA, oral administration may result in suboptimal therapeutic bioavailability, and can even lead to complications such as gastrointestinal injury, liver and kidney failure [96]. Furthermore, because cartilage lacks direct vascularization, only a limited amount of drug molecules can reach the target area. This significantly reduces the effectiveness of treatment. In contrast, injection of drugs directly into the affected joint (intra-articular, IA) can achieve high drug levels in the joint initially [97]. Nevertheless, this approach is often constrained by the rapid elimination of small drug molecules from the joint spaces via synovial capillaries and lymphatics [98]. Therefore, in order to enhance target capacity and extend circulation time, a nano-based drug delivery system has been developed with a large specific surface area and high loading efficiency, and MSC-EVs may be a good choice (Figure 3).

In comparison with synthetic vesicles, EVs have low immunogenicity. Furthermore, EVs offer several advantages over other types of natural vesicles. EVs can effectively incorporate similar exogenous biomaterials, depending on the genetic material and proteins they carry. Additionally, a range of biological fluids, such as blood and breast milk, contain EVs, which demonstrates the body’s tolerance for them. In addition, Evs are also capable of targeting through the enhancement of membrane modification. Therapeutic applications of EVs in bone diseases have been widely reported. To treat bone defects caused by inflammation, bovine milk-derived EV carrying Icariin (ICA) could enhance bone formation by activating the STAT5a and GJA1 genes. As a result, the authors concluded that EV-ICA promotes bone repair more effectively in vivo and in vitro than ICA [99]. Moreover, MSC-Evs were used to carry various RNAs in studies of OA, resulting in improvements in symptoms, protection of cartilage, and inhibition of inflammation. According to Liang et al. [100], exosomes targeted at chondrocytes for miR-140 delivery are a new OA treatment. Exosomes containing chondrocyte-affinity peptides (CAP) can bind to the lysosome-associated membrane glycoprotein 2b protein on their surface, thereby encapsulating miR-140 efficiently and transporting the cargo into chondrocytes, thus alleviating OA progression. As EVs can be retained in the joints for prolonged periods of time, they can also deliver miR-140 deeply inside the cartilage, which indicates that cell-free therapy for OA may be possible.

## 6. Treatment of OA with Engineered MSC-EVs

**Figure 4:**
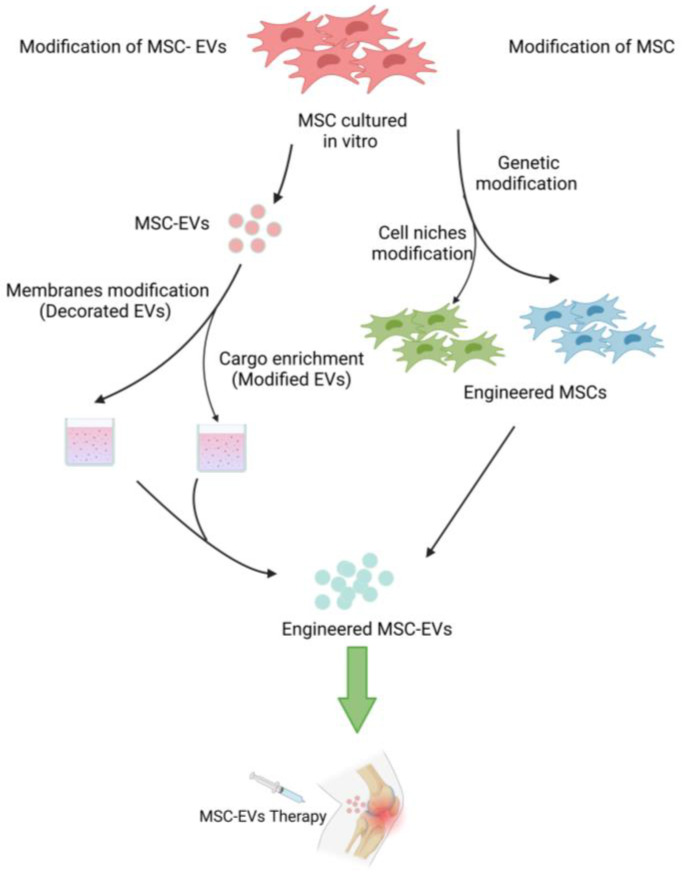
Engineering strategies of MSC-EVs. The diagram illustrates different strategies for producing engineered MSC-EVs to treat OA. Created with BioRender (BioRender.com, 20 February 2023).

While MSC-EV has great promise as an OA therapy, there remain many challenges that need to be overcome before it can be applied in the clinic. MSC-EVs are currently challenged by several limitations, including heterogeneity and immune factors. It has recently been demonstrated that modifying MSC-EVs properly may make them capable of overcoming the limitations of natural MSCs, which may improve EVs’ properties, biodistribution, and bioactivity (Figure 4). To achieve this goal, different engineering techniques are available. They involve either modifying MSC before obtaining engineered MSC-EVs or directly enhancing the engineered MSC-EVs.

### 6.1. Modification of MSC

MSC-EVs tend to inherit traits from MSCs, and donor MSCs can be modified to produce cargo EVs tailored to a particular client’s needs. Researchers can decorate MSCs by manipulating gene transfection or controlling the culture microenvironment, thus regulating the expression of related stimulation factors in a controlled way. 

#### 6.1.1. Genetic Modification 

Exogenous cargos can be packaged into MSC-EVs during the natural biogenesis process, and gene transfection manipulation, via viral or plasmid vectors, is a convenient strategy for enhancing functional cargo expression. Engineered cells have been created using various genetic manipulation approaches. Using these methods, engineered EVs can be produced more efficiently and effectively through miRNAs targeting chondrogenesis. According to Ohno et al. [101], engineered HEK293 cells carrying the let-7a miRNA can release targeted EVs towards epidermal growth factor receptor (EGFR)-expressing breast cancer cells. According to other researchers, elevated expression of miR-140-5p [54], miR-136-5p [91], and miR-338-3p [102] in MSCs and EVs can effectively suppress inflammation, promote ECM synergy and aid in chondrocytes survival. Zhang et al. [87] found that cargo overexpression may cause mRNA degradation, disrupting signal pathways associated with these mRNAs. By genetically manipulating cells, such as MSCs, the possibility of treating cartilage lesions and OA has been expanded.

#### 6.1.2. Cell Micro-Environment Modification

Another effective strategy for enhancing MSC-EV activity is to co-incubate them with biological or chemical factors. Cell niche engineering allows us to build a microenvironment that duplicates the natural cellular environment under controlled physical, chemical, and biological conditions. In 3D cultures, cytokine and miRNA-enriched MSC-derived EVs can be efficiently produced [103]. According to several studies, the differentiation of MSCs into chondrocytes can be efficiently achieved via specific niche design for an MSC-chondrocyte co-culture [104,105]. In light of these findings, we can design an EV niche for treating OA with high-scale engineered EVs derived from 3D-co-cultured engineered MSCs and chondrocytes.

In addition to engineered cells, this method involves manipulating various physical and biochemical factors in an attempt to create large-scale, effective EVs and reconstruct damaged joints. For instance, oxygen concentration is another critical factor related to physical state of MSCs. Compared with normal oxygen concentration (21% O_2_) in vitro culture, MSCs are found in a hypoxic environment in vivo (2–8% or lower oxygen concentration) [106]. As many researchers have suggested, hypoxia may improve MSC-based disease therapy by boosting the biological function and activity of MSCs [107]. To understand the relationship between hypoxic environment and MSC-EVs in OA therapy, Rong et al. [52] use an OA model and perform hypoxic condition in vitro studies. They discovered that hypoxic treatment could promote chondrocyte physical activities including proliferation, migration, and apoptosis suppression, and the miR-216a-5p factor may be involved in promoting the therapeutic effects.

### 6.2. MSC-EV Modification 

Despite the fact that decorative MSC can produce engineered MSC-EVs with their biophysical characteristics, there may be unforeseen effects on EV biogenesis and their biological functions as a result of this process. On the other hand, modified MSC-EVs are generally more efficient. Researchers also can give MSC-EVs different and desired abilities by changing their structures and membranes. Various strategies can be employed to modify the membranes of EVs and improve the drug delivery efficiency to target tissues. Modifying the EVs membrane is one of the common ways. Hydrogels are biocompatible and have a loose and porous structure that lets them act as carriers for EVs. This way, they can keep the cargo in the desired area for longer and regulate its release. Hydrogels incorporating MSC-EVs hold considerable promise as cell-free, sustained delivery platforms for OA treatment. Guan et al. [108] applied GelMA hydrogels with aldehyde-functionalized chondroitin sulfate to BMSC-EVs to form GMOCS- EVs hydrogels. A significant increase in ECM synthesis was observed with GMOCS-EVs hydrogels, which also inhibited inflammation, thereby repairing cartilage damage. Moreover, Tao et al. [109] confirmed the use of an injectable thermosensitive hydrogel containing PDLLA-PEG-PDLLA (PLEL) triblock copolymer loaded with MSC-EVs in the recovery of bone cartilage defects, which remarkably delays the progression of OA.

An alternative method of modification is to increase loading levels of bioactive molecules in MSC-EVs, common methods of which include incubation, both physical and chemical. Using EV membranes as encapsulating surfaces, MSC-EVs are capable of encapsulating hydrophobic drugs [110]. Physical methods such as electroporation, sonication, and extrusion have been used for loading bioactive cargo directly, but these methods may impair the integrity and characteristics of MSC-EVs [111,112,113,114]. Through electroporation, Xu et al. [115] increased the effective concentration of KGN, a small molecule that induces chondrogenesis in MSCs, and the loading efficiency of 40% was achieved compared with 8% obtained with direct co-incubation. This resulted in a significant therapeutic effect on OA progression. Nevertheless, while these methods increase the loading rates of bioactive molecules, their alteration of the structure of MSC-EVs may impair their integrity and capacity, increasing the safety risk.

## 7. Summary and Prospect

As an effective treatment for OA, EVs derived from a variety of MSCs containing miRNAs, lipids, and proteins present great promise. In addition to mimicking the effects of MSCs, MSC-EVs have a lower immune response and a higher safety profile. However, their clinical application remains challenging. The significance of Mesenchymal Stem Cells-Extracellular Vesicles (MSC-EVs) in immune regulation and regeneration has been unequivocally demonstrated. They accomplish this via the regulation of immune responses, the stimulation of effector cell proliferation and migration, as well as the mitigation of apoptosis. Although 15 clinical trials have been registered on ClinicalTrial.gov thus far (Table 3), only one has been completed. Failures in enrollment, funding inadequacy, and various other unforeseeable circumstances could all result in trial failures. Additionally, various studies have shown variations, including the derivation of MSC, EV isolation, and administration details, resulting in heterogeneity.

To start with, the first thing that needs to be discussed is the origin of MSC-EVs. Autologous MSCs are obtainable easily and generally not rejected by the patient’s immune system. However, the isolation, expansion, and release process can take several weeks, and there is a possibility that autologous MSCs may carry potential disease genes from donors [116]. In contrast, research indicates that these cells have demonstrated clinical safety and efficacy, so allogeneic MSCs can be a great candidate with several advantages, including donor selection, varied sources, and low immunogenicity [117]. Recent research has shown MSC-derived extracellular vesicles to be a promising candidate, while EVs may also contain potentially immunogenic proteins such as MHC molecules, leading to detrimental immune responses like those resulting from allogeneic MSCs [118]. It is unclear if MSC-derived exosomes contain MHC molecules or if they may induce allogeneic immune responses, which need further study.

Furthermore, one major limitation of using MSC-EVs as a therapeutic approach for OA is their heterogeneity. EVs derived from different cellular sources under varying conditions can have distinct compositions and functions, leading to variability in therapeutic efficacy. To mitigate this issue, several strategies have been proposed. Firstly, standardized protocols for MSC culture, EV isolation, and characterization can help to ensure consistency in the EV population. Moreover, modifying the MSC culture conditions can help produce EVs with specific properties. Pre-conditioning the MSCs with inflammatory cytokines or growth factors, for example, can induce the secretion of EVs with specific cargo profiles and functions. This can be particularly useful in tailoring EVs to target specific aspects of OA pathology, such as inflammation or cartilage regeneration. Furthermore, engineering MSCs or EVs to express specific proteins or miRNAs can also enhance the therapeutic potential of MSC-derived EVs. For example, overexpressing anti-inflammatory miRNAs in MSCs can enhance the anti-inflammatory effects of the EVs they secrete.

Additionally, it is imperative to develop standardized protocols for the separation and purification of therapeutic EVs. Currently, most studies focus on what happens to chondrocytes after EV treatment, but the molecular mechanisms behind this process remain unknown. To find the most effective medical treatment, it is critical to identify specific targets. New treatment options for OA can be discovered by understanding the mechanisms involved in its development and treatment. Moreover, in recent studies, EVs were combined with biomaterials to deliver drugs to animals locally and proved to be effective in promoting the growth of bone and cartilage. More research is required to compare the effects of EVs on bone diseases when they are given systemically or locally. 

In order to improve the application of MSC-EVs, further exploration will be required in the future. In view of the fact that the current research on EVs in OA, including their role, mechanics, and therapeutic applications, is still in its early stages, a number of questions remain unanswered. As technology advances, we expect MSC-EVs to become a powerful therapeutic option for OA patients.

## Figures and Tables

**Table 1:** Summary of research on the role of MSC-derived EVs in osteoarthritis.

EVs Source	Affected Tissue	Study Type	Disease Model	EVs Dose	Molecular Mechanism	Action Effect	Ref.
hBMSC	Chondrocytes	In vitro	-	1.14 g/mL	Reduce the expression of COX2, ILs and collagenase activity induced by TNF-α while increasing the expression of proteoglycans and type II collagen	Prevent the harmful impact of inflammatory mediators on cartilage homeostasis and facilitate its regeneration	[49]
BMSC	Chondrocytes	In vitro and in vivo	CIOA	-	The overexpression of circHIPK3 resulted in the improvement of IL-1β-induced chondrocyte injury by binding to miR-124-3p	Promote chondrocyte proliferation and migration induction while inhibit chondrocyte apoptosis	[50]
BMSC	Synovial macrophages	In vitro and in vivo	The modified Hulth method	1 μg/mL	Induce synovial macrophages to transform from M1 to M2, decrease the expression of IL-1β, TNF-α, and IL-6 while increasing the expression of IL-10, as well as improving the expression of chondrogenic genes, collagen II, and sox9	Inhibit OA progression	[51]
BMSC	Chondrocyte	In vitro and in vivo	ACLT	200 μg of total protein of sEVs precipitated in 200 μL PBS	The expression of miR-216a-3p was upregulated under hypoxic conditions, leading to the downregulation of JAK2	Enhance cell proliferation and migration, while decreasing apoptosis	[52]
hBMSC	Chondrocytes	In Vitro	-	10 ug/mL	Suppress the pro-inflammatory Erk1/2, PI3K/Akt, p38, TAK1, and NF-κB signaling pathways that are activated by IL-1β	Stimulate the proliferation and migration of chondrocytes affected by osteoarthritis while decreasing their apoptosis rate	[53]
SMSC	Articular chondrocytes	In vitro and in vivo	ACLT	10^11^ exosomes particles/mL	The overexpression of miR-140-5p effectively inhibited the reduction of extracellular matrix secretion by targeting RalA	Enhance proliferation, migration of chondrocytes, and prevent OA	[54]
SMSC	Articular chondrocytes	In vitro and in vivo	ACLT	5 μL SMSC-EV particles per mL	The KDM2A/E2F1/PTTG1 pathway is involved in the beneficial effects of miR-31 on osteoarthritis	Relieve inflammation and cartilage damage in the knee joints	[55]
UMSC	Chondrocyte	In vitro and in vivo	Unilateral cartilage defect	1 mg/mL	Exosomal H19 against miR-29b-3p to upregulate FoxO3	Enhance chondrocyte migration, secretion of matrix, suppression of apoptosis, as well as reduction of senescence	[56]
Infrapatellar fat pad MSCs	Chondrocyte	In vitro and in vivo	DMM surgery	10^10^ particles/mL	MiR100-5p is involved in inhibiting the mTOR-autophagy pathway regulation	The maintenance of cartilage homeostasis can protect the articular cartilage from damage and alleviate gait abnormalities in OA mice	[57]

BMSC bone mesenchymal stem cell, CIOA collagenase-induced osteoarthritis, DMM destabilization of the medial meniscus, EVs extracellular vesicles, OA osteoarthritis, SMSC synovial mesenchymal stem cell, UMSC umbilical cord mesenchymal stem cell, ACLT anterior cruciate ligament transaction.

**Table 2:** Immunomodulatory and cartilage protective effects of MSC-EVs.

	EVs Source	Related Cargo	Effect	Reference
1	BMSCs	MiR-135b	MiR-135b promoted M2 polarization of synovial macrophages through targeting MAPK6, thus improving cartilage damage	[92]
2	hMSCs	-	Activation of AKT/ERK phosphorylation through AMP hydrolysis by MSC exosomes can effectively reduce inflammation and sustain mediate matrix homeostasis.	[91]
3	ADMSCs	-	Reduce inflammatory mediators and restore the ECM by upregulation of annexin A1.	[62]
4	hMSC-miR-92a-3p	-	Exosomal miR-92a-3p regulates cartilage development and homeostasis by directly targeting WNT5A.	[93]
5	hUMSCs	MiR-23a-3p	Improved the migration, proliferation and chondrogenic differentiation of the cells, resulting in the formation of glycosaminoglycan, extracellular matrix and collagen II.	[65]
6	BMSC with kartogenin preconditioning	-	KGN preconditioning endowed BMSC-Exos with stronger chondral matrix formation and less degradation	[94]
7	infrapatellar fat pad (IPFP) MSCs	miR-100-5p	Protect articular cartilage from damage and ameliorate gait abnormality in OA mice via inhibition of mTOR-autophagy pathway.	[57]
8	umbilical cord mesenchymal stem cells (UMSCs)	lncRNA H19	Promote chondrocyte migration, matrix secretion via against miR-29b-3p to upregulate FoxO3 in chondrocytes.	[56]
9	embryonic stem cell-induced mesenchymal stem cells (ESC-MSCs)	-	balance of synthesis and degradation about chondrocyte extracellular matrix (ECM)	[95]

**Table 3:** Clinical trials that use MSC-EVs to treat bone related diseases.

	NCT Number	Title	Conditions	Status	Study Results	Information Provided by (Responsible Party)
1	NCT04173650	MSC EVs in Dystrophic Epidermolysis Bullosa	Dystrophic Epidermolysis Bullosa	Not yet recruiting	No Results Available	Third Affiliated Hospital, Sun Yat-Sen University
2	NCT05078385	Safety of Mesenchymal Stem Cell Extracellular Vesicles (BM-MSC-EVs) for the Treatment of Burn Wounds	Not yet recruiting	Not yet recruiting	No Results Available	Aegle Therapeutics
3	NCT05881668	MSC-EV in Acute-on-Chronic Liver Failure After Liver Transplantation	Liver Failure, Acute on Chronic	Not yet recruiting	No Results Available	Aegle Therapeutics
4	NCT05078385	Safety of Mesenchymal Stem Cell Extracellular Vesicles (BM-MSC-EVs) for the Treatment of Burn Wounds	Burns	Not yet recruiting	No Results Available	Maimónides Biomedical Research Institute of Córdoba
5	NCT05523011	Safety and Tolerability Study of MSC Exosome Ointment	Completed	Not yet recruiting	No Results Available	Paracrine Therapeutics Dermatology Pte. Ltd.
6	NCT05060107	Intra-articular Injection of MSC-derived Exosomes in Knee Osteoarthritis (ExoOA-1)	Osteoarthritis, Knee	Not yet recruiting	No Results Available	Francisco Espinoza, Universidad de los Andes, Chile
7	NCT05216562	Efficacy and Safety of EXOSOME-MSC Therapy to Reduce Hyper-inflammation In Moderate COVID-19 Patients	SARS-CoV2 Infection	Recruiting	No Results Available	Dermama Bioteknologi Laboratorium|Kementerian Riset dan Teknologi/Badan Riset dan Inovasi Nasional, Indonesia
8	NCT05738629	Safety and Efficacy of Pluripotent Stem Cell-derived Mesenchymal Stem Cell Exosome (PSC-MSC-Exo) Eye Drops Treatment for Dry Eye Diseases Post	Dry Eye Disease	Not yet recruiting	No Results Available	Second Affiliated Hospital, School of Medicine, Zhejiang University|Zhejiang University|Hangzhou yuansheng biotechnology Co., Ltd., China.
9	NCT03437759	MSC-Exos Promote Healing of MHs	Macular Holes	Unknown status	No Results Available	Tianjin Medical University
10	NCT04850469	Study of MSC-Exo on the Therapy for Intensively Ill Children	Sepsis|Critical Illness	Withdrawn	No Results Available	Children’s Hospital of Fudan University
11	NCT04388982	The Safety and the Efficacy Evaluation of Allogenic Adipose MSC-Exos in Patients with Alzheimer’s Disease	Alzheimer Disease	Unknown status	No Results Available	Ruijin Hospital|Cellular Biomedicine Group Ltd.
12	NCT05243368	Evaluation of Personalized Nutritional Intervention on Wound Healing of Cutaneous Ulcers in Diabetics	Foot, Diabetic	Not yet recruiting	No Results Available	Maimónides Biomedical Research Institute of Córdoba
13	NCT04798716	The Use of Exosomes for the Treatment of Acute Respiratory Distress Syndrome or Novel Coronavirus Pneumonia Caused by COVID-19	COVID19|Novel Coronavirus Pneumonia|Acute Respiratory Distress Syndrome	Not yet recruiting	No Results Available	AVEM HealthCare
14	NCT04602442	Safety and Efficiency of Method of Exosome Inhalation in COVID-19 Associated Pneumonia	COVID19|SARS-CoV-2 PNEUMONIA|COVID-19	Unknown status	No Results Available	Olga Tyumina|Clinics of the Federal State Budgetary Educational Institution SSMU|Samara Regional Clinical Hospital V.D. Seredavin|State-Financed Health
15	NCT04491240	Evaluation of Safety and Efficiency of Method of Exosome Inhalation in SARS-CoV-2 Associated Pneumonia	COVID19|SARS-CoV-2 PNEUMONIA|COVID-19	Completed	Has Results	State-Financed Health Facility “Samara Regional Medical Center Dinasty”|Clinics of the Federal State Budgetary Educational Institution SSMU|Samara Regional

## Data Availability

Not applicable.
